# Raf-1 Activation Prevents Caspase 9 Processing Downstream of Apoptosome Formation

**DOI:** 10.1155/2011/834948

**Published:** 2010-10-14

**Authors:** Sébastien Cagnol, Anna Mansour, Ellen Van Obberghen-Schilling, Jean-Claude Chambard

**Affiliations:** Institute of Developmental Biology and Cancer, CNRS UMR6543, A. Lacassagne Center, University of Nice Sophia-Antipolis, 33 Avenue Valombrose, 06189 Nice, France

## Abstract

In many cell types, growth factor removal induces the release of cytochrome-c from mitochondria that leads to activation of caspase-9 in the apoptosome complex. Here, we show that sustained stimulation of the Raf-1/MAPK1,3 pathway prevents caspase-9 activation induced by serum depletion in CCL39/ΔRaf-1:ER fibroblasts. The protective effect mediated by Raf-1 is sensitive to MEK inhibition that is sufficient to induce caspase-9 cleavage in exponentially growing cells. Raf-1 activation does not inhibit the release of cytochrome-c from mitochondria while preventing caspase-9 activation. Gel filtration chromatography analysis of apoptosome formation *in cells* shows that Raf-1/MAPK1,3 activation does not interfere with APAF-1 oligomerization and recruitment of caspase 9. Raf-1-mediated caspase-9 inhibition is sensitive to emetine, indicating that the protective mechanism requires protein synthesis. However, the Raf/MAPK1,3 pathway does not regulate XIAP. Taken together, these results indicate that the Raf-1/MAPK1,3 pathway controls an apoptosis regulator that prevents caspase-9 activation in the apoptosome complex.

## 1. Introduction

Apoptosis or programmed cell death is required to maintain cell homeostasis in multicellular organisms [1], its role is essential during embryogenesis as well as in many physiological processes, like immune responses. The components of the apoptotic machinery are expressed in every living cell but remain inactive thanks to environmentally regulated survival signaling pathways [2]. Deregulation of apoptosis control is associated with pathologic processes like neurodegenerative diseases or cancer [3, 4]. Apoptosis execution requires activation of the caspase cascade, which is initiated by two major death-signaling pathways [5]. The extrinsic pathway leads to the activation of caspase 8 after stimulation of cell surface death receptors and the intrinsic pathway, which depends on mitochondrial membrane disruption, leads to caspase 9 activation. Mitochondrial membrane integrity is controlled by the ratio of pro- and anti-apoptotic members of the Bcl2 protein family [6]. The proapoptotic Bcl2 members like Bax Bad or Bim are involved in the release of apoptogenic proteins such as cytochrome c, smac, and Omi/HTrA2 in the cytosol [7]. Cytosolic cytochrome c binds to apoptotic protease activating factor 1 (APAF-1) and induces the formation of an APAF-1-cytochrome c heptamer complex [8] or apoptosome. This event is regulated by the oncoprotein prothymosin *α* [9] and heat-shock proteins [10]. Apoptosome recruits and facilitates the autoproteolytic activation of caspase 9 by homodimerization [11]. Once activated, caspase 9 cleaves and activates effector caspases such as caspase 3 and 7 which in turn cleave numerous cellular substrates, finalizing the process of cell death. Caspase 9 activation is controlled by a putative tumor suppressor PHAP [9] and by the binding of X-linked inhibitor of apoptosis (XIAP) [12]. In turn, XIAP is inhibited by the mitochondrial proapoptotic protein Smac/DIABLO and HtrA2/OMI, when they are released into the cytosol [13].

 In tissue cell culture, continuous activation of growth factor-stimulated survival pathways is required to prevent apoptosis since growth factors withdrawal induces a metabolic arrest [14] that activates the intrinsic pathway of apoptosis and caspase 9. Among the signaling pathways controlled by serum growth factors, the Raf-1/MAPK pathway has been shown to play important roles in cell survival [15]. Knockout studies in mice reveal that *raf*-1 and b-*raf* are involved in developmental cell survival [16]. In human, constitutive activation of MAPK1,3 pathway by Ras or B-Raf oncogenes, is implicated in inhibition of apoptosis in tumor cells, especially in leukemia and melanoma [17, 18]. 

 The MAPK1,3 pathway is involved in the control of Bcl2 family proteins. MAPK1,3 activation has been found to increase the expression of the anti-apoptotic proteins Bcl2, BclXL, and Mcl-1 [19, 20] by transcriptional and posttranslational mechanisms. On the other hand, the MAPK1,3 pathway inactivates proapoptotic members like Bad and Bax by phosphorylation [21] and enhances degradation of Bim by the proteasome [22]. The Raf-1/MAPK1,3 pathway has also been implicated in caspase inhibition downstream of cytochrome c release [23–25]. A potential target of MAPK1,3 is caspase 9 whose activation *in vitro* is inhibited through direct phosphorylation at Thr 125 by MAPK1 [26]. Caspase 9 was shown to be transiently phosphorylated upon MAPK activation in intact cells but there is not yet evidence that this phosphorylation event prevents the processing of the proform during apoptosis induction. In CCL39 ∆Raf-1:ER cells, we and others have shown that sustained activation of MAPK1,3 pathway inhibits matrix detachment and serum deprivation-induced apoptosis [27, 28].

In this study, we further investigated the mechanism by which MAPK1,3 activation blocks serum deprivation-induced caspase 9 cleavage. We show that caspase 9 inhibition requires MEK activity and protein synthesis. This anti-apoptotic effect occurs downstream of cytochrome c release and does not interfere with APAF-1 oligomerization or caspase 9 recruitment into high molecular weight complexes. Furthermore we show that the expression levels of XIAP do not correlate with protein synthesis-dependent caspase 9 inhibition. These results indicate that the Raf-1/MAPK1,3 pathway blocks caspase 9 activation in the apoptosome.

## 2. Material and Methods

### 2.1. Reagents

The MEK inhibitor U0126 was obtained from Promega (Madison, WI), PD98059, emetine, 4-hydroxytamoxifen (4-HT) and the mouse monoclonal anti-activated MAPK (Sigma M8159; 1 : 10,000) were from Sigma (St. Louis, MO). PD184352 was kindly provided by Professor P Cohen (MRC University of Dundee, UK). The monoclonal anti-poly (ADP-ribose) polymerase (anti-PARP) anti-body (Signal Transduction SA-250 1 : 2,000) and caspase 9 peptide substrate Ac-LEHD-pNA were purchased from Biomol (Plymouth Meeting, PA). Other anti-bodies used include rabbit polyclonal anti-MAPK1 (1 : 5,000) [29], mouse monoclonal anti-denaturated cytochrome c (Pharmingen BD biosciences San Jose CA, 556433, 1 : 2,000), rabbit polyclonal anti-active caspase 3 (Cell Signaling Technology Danvers MA, 9661 1 : 200), mouse monoclonal anti-caspase 9 (MBL Woburn MA M054-3 1 : 2,000) and mouse monoclonal anti-XIAP (Transduction Laboratories BD biosciences San Jose CA, 1 : 250).

### 2.2. Cell Culture

CCL39-ΔRaf-1:ER cells (clone S18) [30] Chinese hamster fibroblasts stably express an estradiol-regulated form of oncogenic Raf-1 kinase (plasmids were kindly provided by Dr. M. McMahon, University of California San Francisco Cancer Center, San Francisco, CA). CCL39-ΔRaf-1:ER cells were cultivated in DMEM (Life Technologies, Gaithersburg, MD) without phenol red and supplemented with penicillin (50 U/ml), streptomycin (50 *μ*g/ml), 7.5% fetal calf serum (FCS), and G418 (400 *μ*g/ml).

### 2.3. SDS-PAGE and Western Blot Analysis

Adherent and floating cells were lysed in Laemmli sample buffer, sonicated 15 seconds, and incubated at 65°C for 15 minutes. This protocol, recommended by the manufacturer of the anti-PARP anti-body for detection of PARP cleavage, was used for all immunoblot experiments. Proteins were separated by SDS-PAGE and transferred to PVDF membranes (Millipore Billerica MA). Dry blots were blocked in 3% dry milk (dissolved in water for 10 minutes at room temperature), incubated overnight at 4°C with the primary anti-body, washed 5 minutes with tap water. After blocking again, membranes were incubated 1 hour at 4°C with horseradish peroxidase—(HRP, 1 : 40,000) or alkaline phosphatase—(AP, 1 : 2,000) conjugated secondary anti-bodies (Cell Signaling Technology, Beverly, MA). Immune complexes were detected by autoradiography following enhanced chemiluminescence with SuperSignal (Pierce Thermo Fisher Rockford IL) for HRP or CDP-star (NEB Beverly, MA) for AP.

### 2.4. Fractionation by Gel Filtration

The cell lysates (100,000 g supernatant) were prepared by resuspending cells in a buffer containing 50 mM PIPES/KOH, 2 mM EDTA, 0.1% (w/v) CHAPS, 5 mM dithiothreitol and a protease inhibitor cocktail (Roche). After a freeze/thaw cycle in liquid nitrogen, the lysates were fractionated by size-exclusion chromatography using a fast protein liquid chromatography protein purification system (Amersham Pharmacia Biotech GE Buckinghamshire UK) on an analytical (16/60) Hi Prep S300 Sephacryl high-resolution column (Amersham Pharmacia Biotech). The column was pre-equilibrated with 5% (w/v) sucrose, 0.1% (w/v) CHAPS, 20 mM HEPES, 5 mM dithiothreitol, pH 7.0. All separations were carried out at room temperature. The column was calibrated with gel filtration protein standards from Sigma (thyroglobulin MW = 669,000, ferritin MW = 443,000, *β* amylase MW = 200,000, alcohol dehydrogenase MW = 150,000, bovine serum albumin MW = 66,000 ). 

 Lysates (5 mg of protein) were applied to and eluted from the column at a flow rate of 0.4 ml/min, 500 *μ*l fractions were collected and stored at −70°C. 

### 2.5. Enzymatic Assay for Caspase 9 Activity

Cell lysates (100,00 g fraction of supernatant) were prepared by resuspending cells for 20 minutes at 4°C in buffer containing 0.1% (w/v) CHAPS, 0.5% Nonidet P-40, 50 mM HEPES pH 7.4, 100 M NaCl, 1 mM EGTA, and 10 mM dithiothreitol. Cell lysates (200 *μ*g protein) were incubated with 200 *μ*M caspase 9 peptide substrate Ac-LEHD-*p*NA at 37°C for 1 hour. The *p*NA light emission was quantified using a microtiter plate reader at 405 nm (Labsystems iEMS Reader MF Helsinky Finland).

### 2.6. In Vitro Activation of Caspase 9

Cell lysates were prepared by resuspending cells for 20 minutes at 4°C in buffer A (20 mM HEPES pH 7.4 10 mM KCl, 1.5 mM MgCl_2_, 1 mM EGTA, and 5 mM dithiothreitol) followed by centrifugation at 10,000 g for 20 minutes. Aliquots of the supernatant containing 5 mg/ml protein were frozen in liquid nitrogen and kept at −70°C. Activation of caspase 9 was induced by the addition of 1 *μ*M bovine heart cytochrome c (Sigma St Louis MI) and 1 mM ATP (Fermentas Thermo Fisher Rockford IL) to 20 *μ*l cell lysate and incubated at 20°C, the reaction was stopped by addition of 2% SDS.

### 2.7. Immunofluorescence Assays

Cells were fixed in 3% paraformaldehyde, permeabilized with PBS/0.2% Triton and blocked with PBS/10% FCS. Cells were stained for 1 hour with an anti-cytochrome c monoclonal mouse anti-body (Pharmingen 556432 1 : 200) and with an anti-active caspase 3 rabbit polyclonal anti-body (Cell Signaling 9661 1 : 200). Cells were washed twice with PBS and blocked again with PBS/10% FCS before staining for 1 hour with Alexa-594 coupled anti-mouse anti-body, Alexa-488 coupled anti-rabbit anti-body from Molecular Probes (Eugene, OR). Nuclei were stained for 15 minutes with 50 ng/ml 4′,6-diamidino-2-phenylindole dihydrochloride (DAPI; Roche, Hertforshire, United Kingdom). Fluorescence was observed on a Zeiss inverted microscope (Axiovert 200 M) equipped with a CoolSnap HQ cooled charge-coupled-device camera (Roper Scientifique, Every, France). Image acquisition and analysis was performed using the MetaMorph Imaging System (Universal Imaging Corp. Buckinghamshire UK).

### 2.8. Caspase 9 Expression Vectors

Caspase 9 was cloned in Topo vector (Invitrogen) following RT-PCR amplification from HeLa cell total RNA. PCR primers flanking the coding sequence were designed from the sequence U60521 (genbank) [31] and correspond to nt14–34 AGGCGGCCTGGAGTCTTAGTT and nt1311–1329 ACCCTGCCTTATCTTGCAC. A BamHI restriction site was introduced by PCR at the initiation codon and the BamHI/EcoRI insert was cloned in pCMV-tag 3B (Stratagene) to introduce the tag myc in N-terminal. A potential MAPK1,3 phosphorylation site at Thr 125 was detected using the Phosphobase algorithm at high stringency http://phospho.elm.eu.org/. The threonine was replaced by an alanine using Quick Change mutagenesis kit (Quiagen Hilden Germany). To derive stable cell lines expressing these constructs, the catalytic cysteine (C287) was replaced by a serine. Transfections were performed by the DNA-calcium phosphate coprecipitation method.

### 2.9. D Gel Analysis

Cells were lysed with 1 vol 10 mM Tris, 1 mM EDTA, 0.5% CHAPS containing phosphatase and protease inhibitors. Proteins were precipitated by addition of 3 vol acetone at −20°C and collected at 10000 g for 10 minutes. The pellets were resuspended at 10–20 mg/ml in IEF sample buffer (7 M urea, 2 M thiourea, 2% CHAPS, 40 mM Tris and ampholytes). The first dimension was performed in a Zoom IPG Runner (Invitrogen) on pH 4–7, 7 cm strips. The transfer blots and corresponding autoradiograms were aligned using amido black staining of the PI calibration markers (creatine phosphokinase carbamylyte, Amersham pharmacia Biotech Buckinghamshire UK).

## 3. Results

### 3.1. Raf-1 Activation Prevents Caspase 9 Cleavage upon Serum Withdrawal

**Figure 1:**
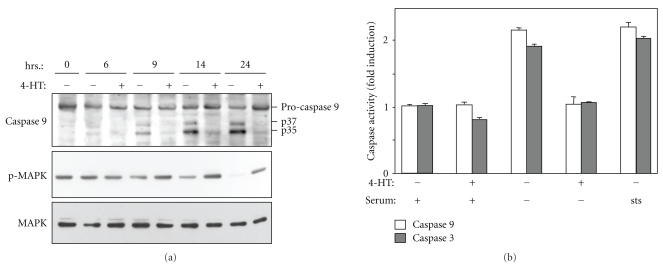
(a) Raf-1 activation inhibits caspase-9 cleavage upon serum withdrawal. CCL39-ΔRaf-1:ER cells treated with or without 4-HT were deprived of serum for the indicated time periods. The cell lysates were analyzed by immunoblotting for caspase-9, phospho-MAPK1,3, and total MAPK1. (b) Raf-1 activation inhibits caspase activity CCL39-ΔRaf-1:ER-cells treated or not with 4-HT were deprived of serum for 14 hours as indicated. sts: the effect of 0.3 mM staurosporin is shown as a comparison. Caspase 9 activity was measured using the peptide LEHD-pNA as a substrate and caspase 3 activity using DEVD-pNA. The mean values ± SEM were calculated from at least two separate experiments performed in triplicate.

In exponentially growing CCL39-ΔRaf-1:ER cells, sudden growth factor deprivation triggers apoptosis. Although interactions with components of the extracellular matrix provide anti-apoptotic signals to these adherent fibroblasts, serum removal for 24 hours results in more than 50% cell death, as determined by propidium iodide staining (not shown). CCL39-∆Raf-1:ER cells express the fusion protein ∆Raf-1:ER, therefore, addition of 4-hydroxytamoxifen (4-HT) results in the selective and persistent activation of Raf-1 signaling. 4-HT addition to serum-deprived cells leads to a complete inhibition of apoptosis (data not shown, [27, 28]). As shown in Figure 1(a), serum removal gradually decreased the levels of phosphorylated MAPK1 and 3 (also known as ERK1 and 2) and this was accompanied by the appearance of the p35 and p37 cleavage products of pro-caspase 9. The presence of 4-HT maintained phosphorylation of MAPK1,3 and completely inhibited caspase 9 cleavage. 

 Consistent with the fact that procaspase 9 cleavage is generally associated with the generation of the active form of caspase 9, we observed an increase in caspase 9 activity in cell extracts prepared 14 hours after serum removal and this activity was significantly inhibited in the presence of 4-HT (Figure 1(b)). Caspase 3 is known to be a direct substrate of caspase 9, accordingly its activation was also found to be repressed upon Raf-1 stimulation (Figure 1(b)). These results indicate that sustained Raf-1 activation inhibits caspase 9 cleavage and activity following serum removal thereby preventing apoptosis to proceed.

### 3.2. Caspase 9 Activation Is Inhibited by the MEK/MAPK1,3 Pathway

**Figure 2:**
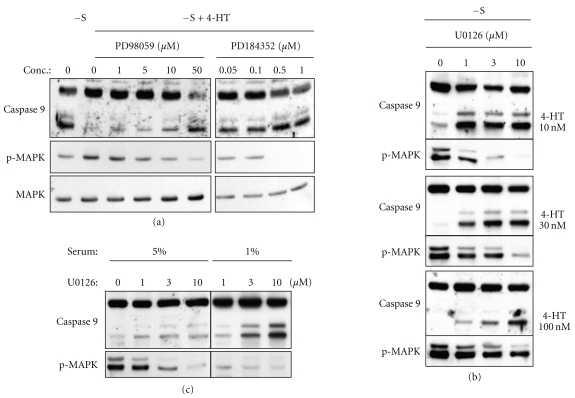
Raf-1-mediated caspase-9 inhibition depends on MEK activity. (a) CCL39-ΔRaf-1:ER cells were serum deprived for 24 hours in the absence or presence of 1 *μ*M 4-HT. The indicated concentrations of the MEK inhibitors PD98059 and PD184352 were added 30 minutes before 4-HT addition. The cell lysates were analyzed by immunoblotting for caspase-9, phospho-MAPK1,3, and total MAPK1. (b) Dose response for U0126 at different 4-HT concentrations. (c) Exponentially growing CCL39-ΔRaf-1:ER cells in the presence of 5% or 1% fetal calf serum were treated for 24 hours with increasing concentrations of U0126.

To determine the role of Map kinases in the inhibition of caspase 9 cleavage, 4-HT-treated cells were incubated with increasing concentrations of the specific MEK1 inhibitors U0126, PD98059 and PD184352. All of these pharmacological agents prevented Raf-1-induced MAPK activation and reversed the cleavage of caspase 9 (Figure 2(a)). 4-HT-induced Raf-1:ER activation is dose-dependent, the dose-response experiment shown in Figure 2(b) clearly indicates a close relationship between MAPK phosphorylation and caspase 9 cleavage. It should be noted that in exponentially growing cells, the inhibition of MEK by U0126 was sufficient to induce a weak cleavage of caspase 9 (Figure 2(c)), which is more pronounced at lower serum concentration. Thus, we can conclude that the inhibition of caspase 9 cleavage by Raf-1 depends on MEK activity and MEK activity contributes permanently to the protection against caspase 9 activation provided by serum.

### 3.3. Caspase 9 Inhibition Requires Protein Synthesis and Persistent MEK Activity

**Figure 3:**
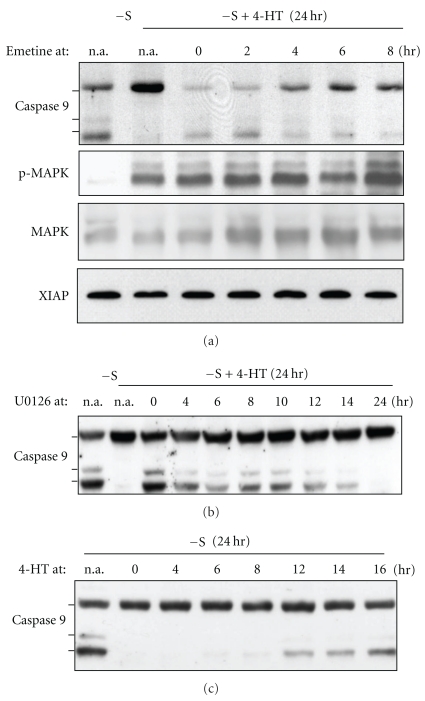
(a) Raf-1-mediated protection of caspase 9 cleavage requires protein synthesis. CCL39-ΔRaf-1:ER cells were deprived of serum for a total time of 24 hours in the presence or absence of 1 *μ*M 4-HT. The protein synthesis inhibitor emetine (20 *μ*M) was added at the indicated time point. The cell lysates were analyzed by immunoblotting for caspase-9, phospho-MAPK1,3, total MAPK1 and XIAP. (b) and (c) time course of Raf-1-mediated caspase 9 inhibition. (b) CCL39-ΔRaf-1:ER cells were deprived of serum for a total time of 24 hours in the presence of 50 nM 4-HT. 10 *μ*M U0126 was added at the indicated time after serum withdrawal. (c) Cells were deprived of serum for 24 h; 4-HT (1 *μ*M) was added at the indicated time after serum withdrawal. The cell lysates were analyzed by immunoblotting for caspase-9.

We then determined if the protective effect of Raf-1 would require de novo protein synthesis. Because cycloheximide has been shown to completely prevent serum deprivation-induced apoptosis in CCL39-derived lines (our unpublished observations and [28]), we analyzed the effect of emetine, another protein synthesis inhibitor. As shown in Figure 3(a), addition of emetine at the same time as 4-HT (lane 3, emetine at time 0) completely blocked Raf-1-mediated caspase 9 inhibition, without affecting Raf-1-induced phosphorylation of MAPK. Raf-1-mediated protection was still ineffective when emetine was added two hours after 4-HT stimulation and was gradually restored when emetine was added later. Addition of emetine 8 hours after Raf-1 activation (last lane Figure 3(a)) did not affect inhibition of caspase 9 cleavage anymore since only full-length procaspase 9 is detected. These results indicate that Raf-1-mediated caspase 9 inhibition requires protein synthesis and that complete protection is achieved after 6–8 hours accumulation of a caspase 9 inhibitor. XIAP is one of the potential inhibitors of caspase 9 downstream cytochrome c release. Thus, we investigated the expression of XIAP following 4-HT treatment of CCL39-ΔRaf-1:ER cells and found that XIAP expression was not affected by Raf-1 activity or by emetine treatment (Figure 3(a)). These results indicate XIAP is not the emetine-sensitive inhibitor of caspase 9.

 Caspase 9 processing have been shown to be regulated *in vitro* by direct MAPK1,3 phosphorylation [26], to determine if Raf-1/MAPK-mediated protection of caspase 9 cleavage in intact cells was only a posttranslational mechanism we determined the time course of Raf-1 action.  Figure 3(b) shows that delayed addition of U0126 after 4-HT as a mean to terminate Raf-1/MAPK action resulted in caspase 9 cleavage. When U0126 was added 12, 10, or 8 hours after 4-HT and the experiment terminated at 24 hours, the cells remained unprotected for 12, 14, and 16 hours, respectively. Under these conditions, the extent of caspase 9 cleavage was similar to that in cells that were maintained unprotected for the same times because of delayed addition of 4-HT (Figure 3(c)) This result indicates that 4HT preincubation is not sufficient to prevent caspase 9 cleavage. Thus, caspase 9 protection requires both protein synthesis and continuous Raf-1 signaling.

### 3.4. Raf-1/MAPK Activation Induces Posttranslational Modifications of Caspase 9

**Figure 4:**
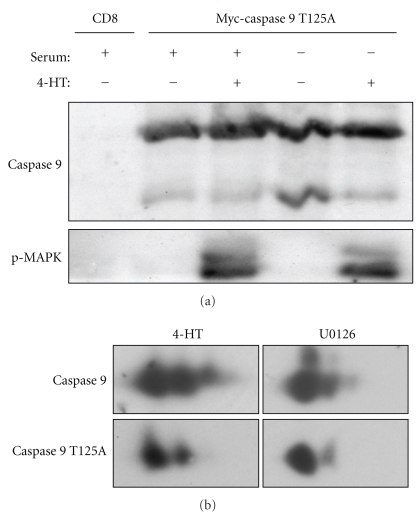
Mutation of threonine 125 does not prevent caspase 9 phosphorylation and cleavage inhibition. (a) CCL39-ΔRaf-1:ER cells were transfected with the indicated construct (CD8 is used as a transfection control) and 48 hours later were deprived of serum in the presence or absence of 1 *μ*M 4-HT for 24 hours. The cell lysates were analyzed by immunoblotting for myc and phospho-MAPK1,3. (b) CCL39-ΔRaf-1:ER cells stably expressing myc-tagged C287S (catalytically inactive) caspase 9 or C287S-T125A caspase 9 were incubated for 2 hours with 1 *μ*M 4-HT or 10 *μ*M U0126 in serum-free medium. Total cells were lysed in 2D sample buffer containing 6 M urea, resolved by two-dimensional gel electrophoresis, and analyzed by immunoblotting for myc.

The above-mentioned experiments suggest the involvement of posttranslational mechanisms in the protection of caspase 9 cleavage, we thus investigated the role of the recently identified MAPK1,3 phosphorylation site T125 present on caspase 9.  Figure 4(a) shows the transient expression of a myc tagged mutated form of caspase 9 in which T125 is substituted by the nonphosphorylatable residue alanine. Serum withdrawal-induced cleavage of caspase 9 T125A was totally prevented by 4-HT addition. This results indicates that phosphorylation of T125 is not involved in Raf-1/MAPK protection of caspase 9 cleavage. 

 In addition, two-dimensional electrophoresis analysis of catalytically inactives capase 9 and mutant caspase 9 after Raf-1 stimulation (Figure 4(b)) revealed that caspase T125A was still able to shift in response to 4-HT addition. This result suggests the presence of an additional phosphorylation site on caspase 9. In the presence of 4-HT, caspase 9 clearly showed an additional spot indicating that indeed T125 is phosphorylated in response to 4-HT. Both sites were sensitive to U0126. 

### 3.5. Raf-1 Activation Does Not Prevent Cytochrome c Release

**Figure 5:**
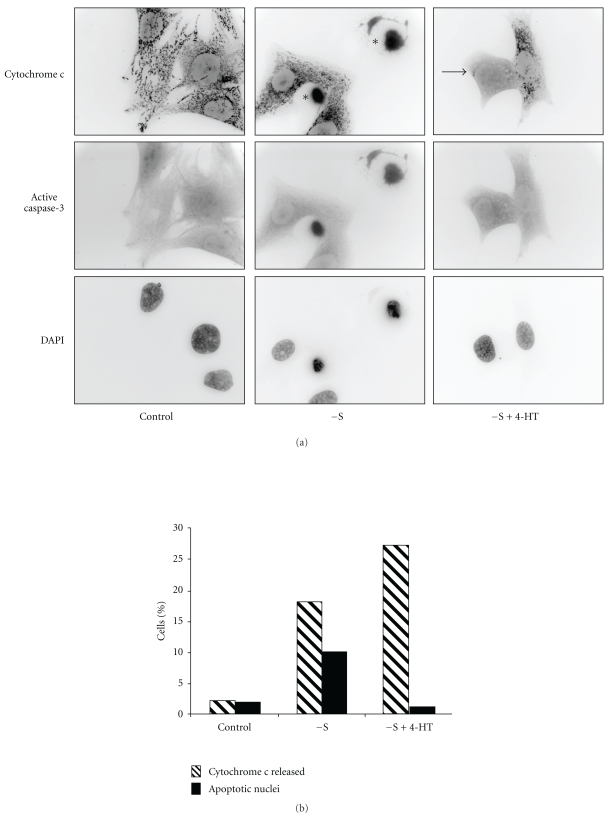
(a) Raf-1 activity does not prevent cytochrome c release. CCL39-ΔRaf-1:ER cells were deprived of serum for 14 hours in the presence or absence of 4-HT. Fixed and permeabilized cells were stained for cytochrome c and active caspase-3. Nuclei were stained with DAPI. Arrows show cells with cytochrome c released. (b) Quantification of Figure 5(a): Cytochrome c release and apoptotic nuclei were quantified from 200 adherent cells in triplicates.

The release of cytochrome c from mitochondria into the cytosol is an important cellular event leading to caspase 9 processing. We, therefore, examined the distribution of cytochrome c following serum removal in control and 4-HT-treated cells by immunofluorescence analysis. As shown in Figure 5(a), control cells presented a typical punctuate staining of cytochrome c corresponding to its mitochondrial localization. After 14 hours of serum removal, 20% of the cells exhibited a rounded morphology (cell marked with asterisk on Figure 5(a)). Only the cells that remained adherent were examined, and since apoptosis induces cell detachment, these values are only indicative of the cell population status at a given time point. Rounded cells presented a diffuse cytochrome c staining and an intensification of active caspase 3 labeling. Since caspase 3 is a direct caspase 9 substrate, the presence of active caspase 3 is indicative of caspase 9 activation. Nuclear condensation was detected in 50% of these cells, indicating that half the cells which released cytochrome c, completed apoptosis 14 hours after serum withdrawal. Interestingly, although 4-HT treatment inhibited caspase 3 activation and nuclear condensation after serum removal, it did not interfere with cytochrome c release (cell marked with arrow on Figure 5(a)). Rather, an increase in cells exhibiting cytoplasmic cytochrome c was detected in 4-HT-treated cells compared to nontreated cells (Figure 5(b)); this finding indicates that Raf-1/MAPK prevents apoptosis to proceed in the presence of cytosolic cytochrome c. The activation of Raf-1 did not perturb mitochondrial localization in control, serum-stimulated cells (not shown).

**Figure 6:**
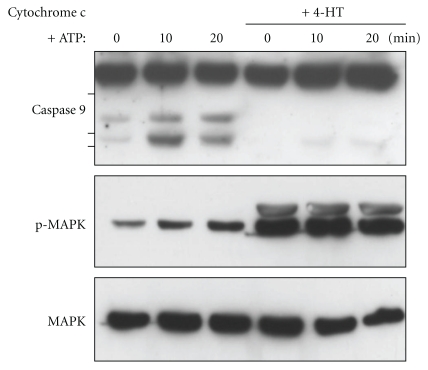
Raf-1 activation prevents cytochrome c/ATP-mediated activation of caspase 9. CCL39-ΔRaf-1:ER cells were deprived of serum for 9 hours in the presence or absence of 4-HT. The cells were lysed in hypotonic buffer and the cell lysates were incubated with 1 *μ*M cytochrome c and 1 mM ATP for the indicated times. The cell lysates were analyzed by immunoblotting for caspase-9, phospho-, and total MAPK1,3.

 To confirm that caspase 9 activation can be inhibited in the presence of cytosolic cytochrome c, we performed *in vitro* assays. Cytosolic extracts from cells deprived of serum for 9 hours in the presence or absence of 4-HT were incubated with 1 *μ*M cytochrome c and 1 mM ATP [32]. Incubation was performed at 20°C in order to avoid the rapid inactivation of MAPK observed at 37°C (not shown). As can be seen in Figure 6, cytochrome c/ATP addition induced caspase 9 cleavage in serum-deprived cell extracts but not in extracts from 4-HT treated cells. A similar result was obtain upon electroporation of purified cytochrome c in intact cells that were preincubated with 4-HT (see Figure S1 in Supplementary material available online at doi:10.1155/2011/834948). These results further demonstrate that Raf-1 activation inhibits caspase 9 cleavage downstream of mitochondrial cytochrome c release.

### 3.6. Raf-1 Activation Does Not Prevent Apoptosome Formation

**Figure 7:**
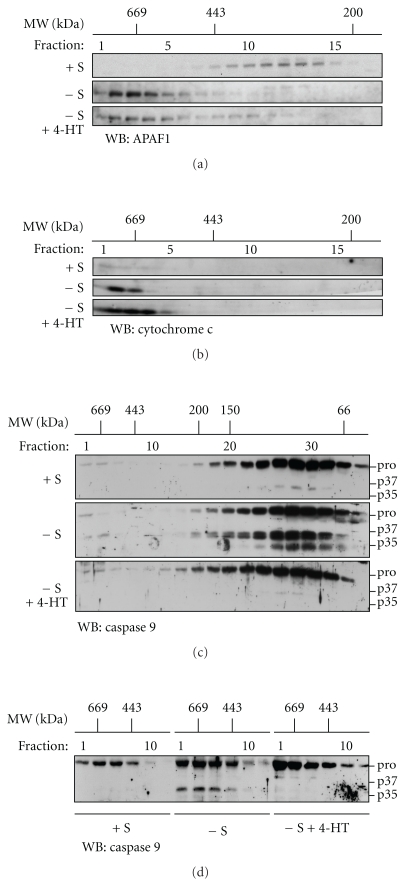
(a) and (b): Raf-1 activity does not interfere with apoptosome formation. CCL39-ΔRaf-1:ER cells growing in 7.5% FCS (control) were deprived of serum (−S) for 14 hours in the presence or absence of 4-HT. The lysates were separated by gel filtration chromatography on a sephacryl S300 column previously calibrated with the indicated molecular weight standards. Samples from high molecular weight fractions were diluted with 4X Laemmli buffer and were analyzed by immunoblotting for the presence of APAF-1 (a) and cytochrome c (b). (c) and (d): recruitment of caspase 9 in the apoptosome. (c) The conditions are the same as in Figures 7(a) and 7(b); samples are a pool of 2 fractions and were analyzed by immunoblotting for caspase 9. pro: procaspase 9, 47 kd. p37, p35: cleaved caspase 9. (d) Recruitment of caspase 9 in high molecular weight fractions. Detailed analysis of high molecular weight fractions from Figure 7(c).

In the cytosol of apoptotic cells, binding of cytochrome c to APAF-1 leads to its oligomerization and recruitment of procaspase 9 into a high molecular weight complex known as the apoptosome [8]. We examined whether Raf-1 activation inhibited procaspase 9 activation in the cells by blocking formation of the apoptosome. To do so size exclusion chromatography was performed on cytosolic extracts of CCL39-ΔRaf-1:ER cells followed by Western blot analysis of the different apoptosome components. Results in Figure 7(a) show that in lysates from control cells in presence of serum, APAF-1 was located in fractions corresponding to *M*_*r*_ of ∼200 kDa–400 kDa. In lysates from serum-deprived cells, the APAF-1 signal shifted to fractions corresponding to approximately *M*_*r*_ of ∼700 kDa, consistent with the formation of an active apoptosome complex *in vivo* [33]. In extracts from serum-stimulated cells, cytochrome c was not detected in fractions containing high molecular weight complex. However, following serum removal, cytochrome c coeluted with APAF-1 in high molecular weight fractions (Figure 7(b)). Raf-1 activation does not interfere with the relocalization of APAF-1 and cytochrome c into high molecular weight complexes upon serum withdrawal (Figures 7(a) and 7(b)), these results suggest that Raf-1 activation does not interfere with the apoptosome formation. The presence of cytochrome c in the high molecular weight fractions also confirm that Raf-1 activation does not block cytochrome c release. We then determined if caspase 9 present in living cells was recruited into the apoptosome (Figure 7(c)) and found that under all conditions, most of the caspase 9 signal was detected in fractions corresponding to *M*_*r*_ of ∼60 kDa to 150 kDa. This in contrast to results obtained after activation in cell extracts where the cleaved caspase 9 is mainly present in the high molecular weight fractions (see Supplementary Figure S2) and indicates that the active caspase 9 is rapidly released from the apoptosome *in vivo*. However, a small caspase signal could be detected in the high molecular weight fractions that could be enhanced by concentrating the eluat. As shown in Figure 7(d), a higher resolution of high molecular weight fractions revealed the presence of caspase 9. Some procaspase 9 was found in the ∼700 kDa fractions of serum containing cell, while its amount increased notably in high molecular weight fractions from serum-deprived cells. The ∼700 kDa fractions from serum-starved cells also contained the cleaved form of caspase 9. In 4-HT-treated serum-deprived cells, a large amount of procaspase 9, but not cleaved caspase 9, was found in the ∼700 kDa fractions. These results indicate that in intact CCl39 cells, the majority of caspase 9 is not associated with APAF-1 and that Raf-1 activity, while preventing caspase 9 cleavage, does not inhibit the pool of caspase 9 that is transiently recruited into the apoptosome. 

 Altogether, these results indicate that the Raf-1/MAPK1,3 pathway controls the cleavage of caspase 9 within the apoptosome.

## 4. Discussion

Growth factor deprivation is often linked to activation of the mitochondrial pathway of apoptosis because overexpression of Bcl2 prevents caspase activation. However, very few reports have documented caspase 9 activation in intact cells. In this paper, we clearly show that caspase 9 is activated and cleaved following serum deprivation of exponentially growing CCL39 cells. Activation of Raf-1:ER by 4-HT leads to the specific activation of the MEK/MAPK1,3 signaling module [27] and prevents caspase 9 activation. A panel of MEK inhibitors inhibits the effects of Raf-1:ER. Since U0126 and PD98059 also inhibit MEK5/ERK5, a pathway involved in Raf-1 signaling [34], we used PD184161 a specific MEK1,2 inhibitor [35] at low concentrations [36] to show that the MEK/MAPK1,3 pathway mediates caspase 9 inhibition. Treatment of exponentially growing CCL39 cells by MEK inhibitor was sufficient to cause the appearance of the p35 and p37 processed caspase 9 products. This finding suggests that MEK activity participates in serum-mediated cell protection and that high levels of MAPK activity are continually required to maintain caspase 9 inactive. Serum deprivation of CCl39 cells has often been used to study cell cycle regulation [37]. To arrest CCL39 cells in G0/G1 and limit cell death upon serum deprivation, cells were usually grown to confluency during several days, the progressive limitation in nutrients resulting in an adaptation of cells to growth factor deprivation. Growth factor deprivation affects nutrient utilization and induces a metabolic arrest that results in apoptosis [14]. It is thus possible that sudden growth factor deprivation has a more pronounced effect in actively growing cells than in quiescent cells with limited nutrient needs. Indeed, most quiescent cells in serum-deprived medium are alive and can reenter the cell cycle, while about 65% of an actively growing cell population dies upon serum removal. This observation might be relevant to tumor cell biology since tumor cells need to maintain a high metabolic rate with limited survival factors. Pharmacological inhibition of MEK activity has been shown to induce cell death in exponentially growing cell lines derived from melanoma [38], leukemia [17], fibrosarcoma, and renal carcinoma [39]. It remains to be determined if MEK inhibitors would induce caspase 9 cleavage in tumor cells.

 Our immunofluorescence and *in vitro* caspase 9 activation assays show that Raf-1-mediated inhibition of caspase 9 occurs at a postcytochrome c release level. The ability of MAPK pathway to prevent apoptosis progression following cytochrome c release has been described in several other cell models. For example, ∆Raf1:ER activation inhibits cell detachment-induced apoptosis in MDCK cells [40] and MEK inhibits caspase 3 activation in serum-deprived Rat1 fibroblasts [23] or in etoposide-treated small cell lung cancer cells [24]. Moreover, MEK activity also blocks cytochrome c-induced caspase 3 activity in Xenopus and Rat1 fibroblast cell free extracts [23, 41]. *In vivo*, acidic pH induced a very similar mechanism of inhibition of caspase 9 processing without impaired cytochrome c release or APAF-1 interaction [42]. NGFs have been shown to mediate neuron resistance to apoptosis in response to cytochrome c microinjection in the cytosol [43]. This observation led to the notion of a “competence to die” induced by growth factor withdrawal; our results indicate that Raf-1/MAPK inactivation would render cells competent to respond to cytosolic cytochrome c and then to die. 

 There is a large body of evidence indicating that MAPK1,3 signaling is involved in the regulation of mitochondria homeostasis. For instance, p90 rsk phosphorylates and inactivates the proapoptotic protein, Bad [21, 44] while direct phosphorylation of the anti-apoptotic protein Bcl2 by MAPK1,3 results in its stabilization and enhanced cell survival [19]. These findings indicate that the MAPK1,3 pathway inhibits apoptosis by preventing cytochrome c release, an hypothesis that has not been directly documented. Indeed, CCL39 cells are sensitive to the anti-apoptotic properties of Bcl2 family proteins because stable expression of BclXl prevents caspase activation upon anchorage and serum withdrawal (our unpublished observations). Thus, under our stringent conditions of apoptosis induction, the putative control of mitochondrial integrity by MAPK1,3 is not efficient enough to prevent cytochrome c release. In CCL39 ∆Raf1:ER cells, Cook and colleagues clearly demonstrated that MAPK-induced degradation of Bim by the proteasome resulted in Bax inactivation [28, 45]. However, downregulation of Bim has not been shown to inhibit cytochrome c release in CCL39 cells, an event that could be regulated by other MAPK-insensitive BH3-containing proteins. 

 Our analysis of apoptosome by gel filtration chromatography indicated that the protective effect of MAPK1,3 pathway does not interfere with APAF-1 multimerization or caspase 9 recruitment. However, even in apoptotic conditions, most of the caspase 9 was located in low molecular weight fractions. Most of the publications illustrating the recruitment of caspase 9 in the apoptosome are performed *in vitro* [46] in the presence of exogenously added cytochrome c and ATP and it is not known how much caspase 9 is associated with multimerized APAF-1 *in vivo*. Since present models of caspase 9 activation indicate that only a minor portion of purified caspase 9 is found in the active dimeric form [12] it is perhaps not surprising to find little caspase 9 in high molecular weight complexes *in vivo*. However, when Raf-1/MAPK is activated there is a clear increase in the recruitment of the procaspase 9 to the 700 kDa complex. Recruitment of procaspase 9 is thought to favor a homodimerization step where a monomer provides, the structural constraints required for the constitution of a functional catalytic cleft [47]. This subsequently results in the autoproteolysis of the link between the large and the small catalytic subunits. Since caspase 9 is not cleaved in 4-HT-treated cells, we can conclude that Raf-1 activation is likely to interfere with the formation of an active dimer. 

 Our results indicate that protein synthesis is required for caspase 9 inhibition. Among the potential inhibitors of the apoptosome, heat-shock proteins are not likely to be involved in Raf-1-induced caspase 9 inhibition, because they interfere either with APAF-1 oligomerization or caspase 9 recruitment [48, 49]. Similarly, an other negative regulator of apoptosis, the oncoprotein prothymosin *α* is not a likely mediator of Raf-1 effects because it prevents apoptosome formation [9] however, the same study identified PHAP as positive regulator of caspase 9 activation in the apoptosome. It is not yet known whether proteins of the PHAP family are required for caspase 9 activation in intact cells and then would be regarded as potential targets of Raf-1/MAPK activity. The major inhibitor of caspase 9 is XIAP. XIAP inhibits the catalytic activity of caspase 9 by using its BIR3 domain to heterodimerize with a caspase 9 monomer through the same interface that is required for the caspase 9 homodimerization [12]. However, caspase 9/XIAP interaction also requires the cleavage of caspase 9 because the newly generated small subunit N-terminal stabilizes the interaction [50]. Since caspase 9 is not cleaved in the presence of activated Raf-1, a stable interaction between caspase 9 and XIAP is not expected, however, overexpression of XIAP by inhibiting newly generated cleaved caspase 9 would prevent further autoprocessing of procaspase monomers In mammals, MAPK activation has been associated with the increase of XIAP expression in melanoma cells [51], monocytes [52] and Jurkat cells [53]. In small-cell lung cancer cells, Downward and colleagues have demonstrated that FGF2 inhibits etoposide-mediated caspase 3 activation downstream cytochrome c [24]. It was found that MAPK1,3 activation by FGF2 inhibits the release of Smac from mitochondria. Since Smac triggers the proteasomal degradation of XIAP, MAP activation resulted in XIAP stabilization. In our hands, XIAP was not induced following Raf-1 activation, as it would be expected if Smac were retained in the mitochondria. Moreover, XIAP levels were insensitive to emetine, an indication that XIAP is not the emetine sensitive anti-apoptotic protein required for Raf-1-mediated caspase 9 protection. We cannot exclude that Raf-1 activity up regulates XIAP anti-apoptotic effect by inducing a cofactor that could enhance caspase 9/XIAP interaction; however, we were not able to coimmunoprecipitate caspase 9 and XIAP in cell extracts from 4-HT treated cells (data not shown).

 Clarke and colleagues reported that MAPK pathway directly controls caspase 9 activation *in vitro* by phosphorylation on its residue Thr125. Although Thr125 phosphorylation of caspase 9 through a MEK-dependent mechanism occurs in PMA treated HeLa cells [26], it has not yet been linked with cell survival. In serum-deprived CCL39 cells expressing a human caspase 9 mutated on its phosphorylation site (caspase 9 T125A), we show that MAPK activation is still able to inhibit the cleavage of this mutant. Thus, it may be argued that phosphorylation of caspase 9 on threonine 125 is not involved in MAPK-mediated caspase 9 inhibition in serum-deprived cells. However, we cannot exclude that the inhibition of endogenous caspase 9 by phosphorylation on T125 interferes with the cleavage of the mutated form within heterodimers despite the fact that caspase 9 T125A was overexpressed by transient transfection. Our 2D gel analysis indicates that caspase 9 T125A still shifts in response to Raf-1 activation, an indication that it is phosphorylated on another residue. We can exclude S196 a site phosphorylated by AKT [54] because this site present on human caspase 9 is not conserved in rodents [55] and Raf-1:ER does not activate Akt in CCl39 cells [27]. Caspase 9 is also phosphorylated by PKA [56], but since PKA is not inhibited by U0126 [57], it is not likely to be involved because U0126 totally prevents caspase shifts on 2D gels. However, we cannot exclude that this MEK-dependent charge shift results from the synthesis or processing of an autocrine ligand [58] that does not activate the PI3K pathway [27], yet activates other sigalling pathway leading to caspase 9 phosphorylation (reviewed in [59]).

 The role of caspase 9 phosphorylation remains to be clarified, since procaspase 9 activation requires an obligatory dimerization step provided by homophilic contact between the catalytic subunits, this region of the caspase could be a site of interaction with a putative inhibitor of dimerization or a site of direct phosphorylation. 

## Supplementary Material

S1: Raf-1 activation prevents caspase 9 cleavage upon cytochrome c electroporation.
S2: Caspase 9 recruitment in the apoptosome following activation of cell extracts with cytochrome c and ATP is prevented by Raf activation.
S3: Raf-1 “kinase-dead” does not activate MAPK nor prevents caspase 9 cleavage.

## Abbreviations

FCS: Fetal calf serum
MAPK: Mitogen-activated protein kinases 1, 3
4-HT: Hydroxytamoxifen.
