# Supplementary material for: Raf-1 Activation Prevents Caspase 9 Processing Downstream of Apoptosome Formation
Source: J Signal Transduct. 2010 Oct 14;2011:834948. doi: 10.1155/2011/834948 (PMC3100593; doi:10.1155/2011/834948)
Supplement: Supplementary file 1 — S1: Raf-1 activation prevents caspase 9 cleavage upon cytochrome c electroporation. S2: Caspase 9 recruitment in the apoptosome following activation of cell extracts with cytochrome c and ATP is prevented by Raf activation. S3: Raf-1 “kinase-dead” does not activate MAPK nor prevents caspase 9 cleavage. [file 834948.f1.pdf]

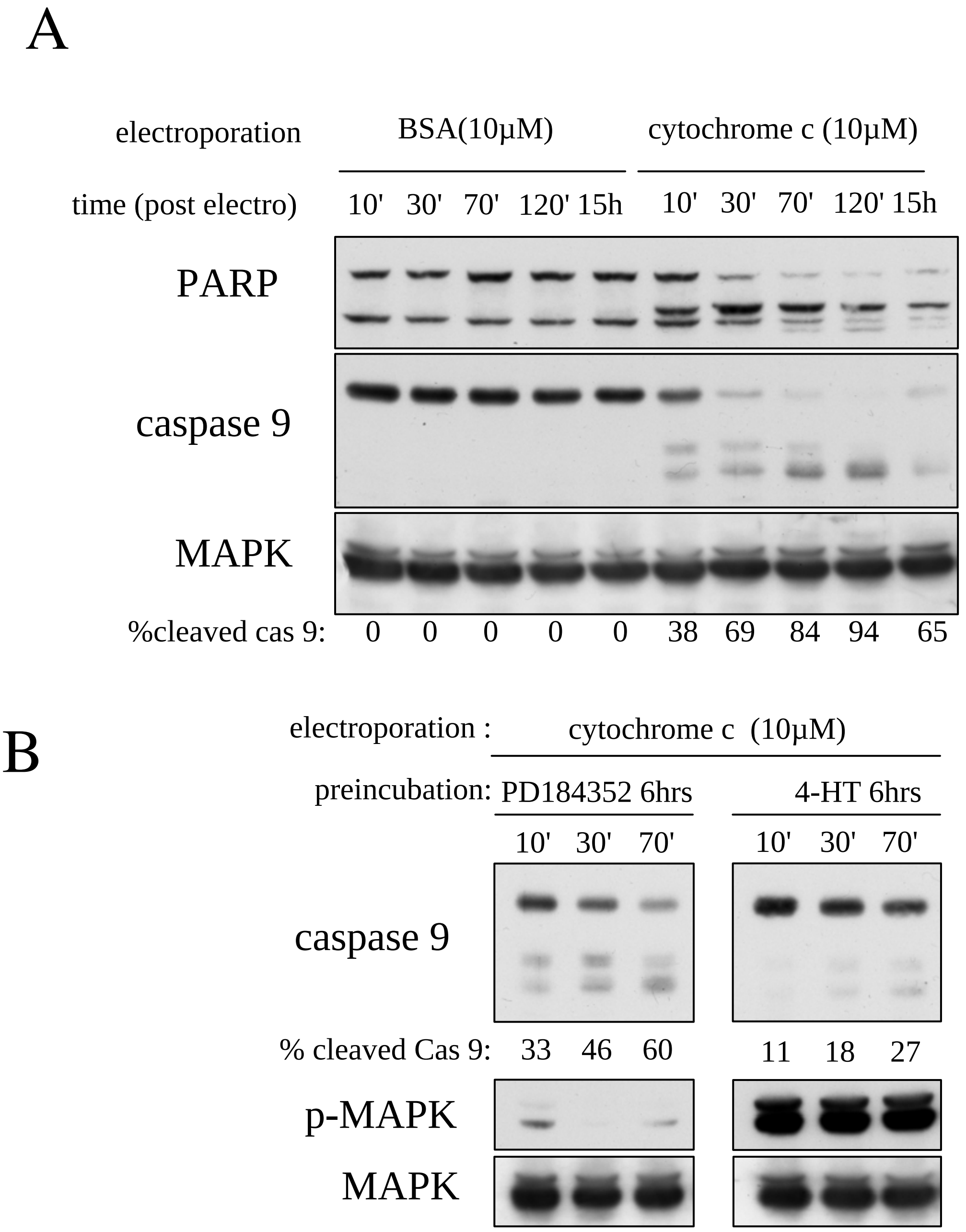

Figure S1: 4-HT preincubation prevents caspase 9 cleavage upon cytochrome c electroporation.

A, 10^6 exponentially growing CCL39-ΔRaf-1:ER cells were trypsinized and resuspended in PBS with the indicated concentration of cytochrome c or BSA, electroporated and allowed to recover in complete medium for the indicated time and lysed with 2%SDS. . The cell lysates were analyzed by immunoblotting. PARP cleavage is indicative of caspase activity.

B, Same as in A, exept that the cells (in serum containing medium) were preincubated with a MEK inhibitor or 4-HT for 6 hours
