# Supplementary material for: Raf-1 Activation Prevents Caspase 9 Processing Downstream of Apoptosome Formation
Source: J Signal Transduct. 2010 Oct 14;2011:834948. doi: 10.1155/2011/834948 (PMC3100593; doi:10.1155/2011/834948)
Supplement: Supplementary file 2 [file 834948.f2.pdf]

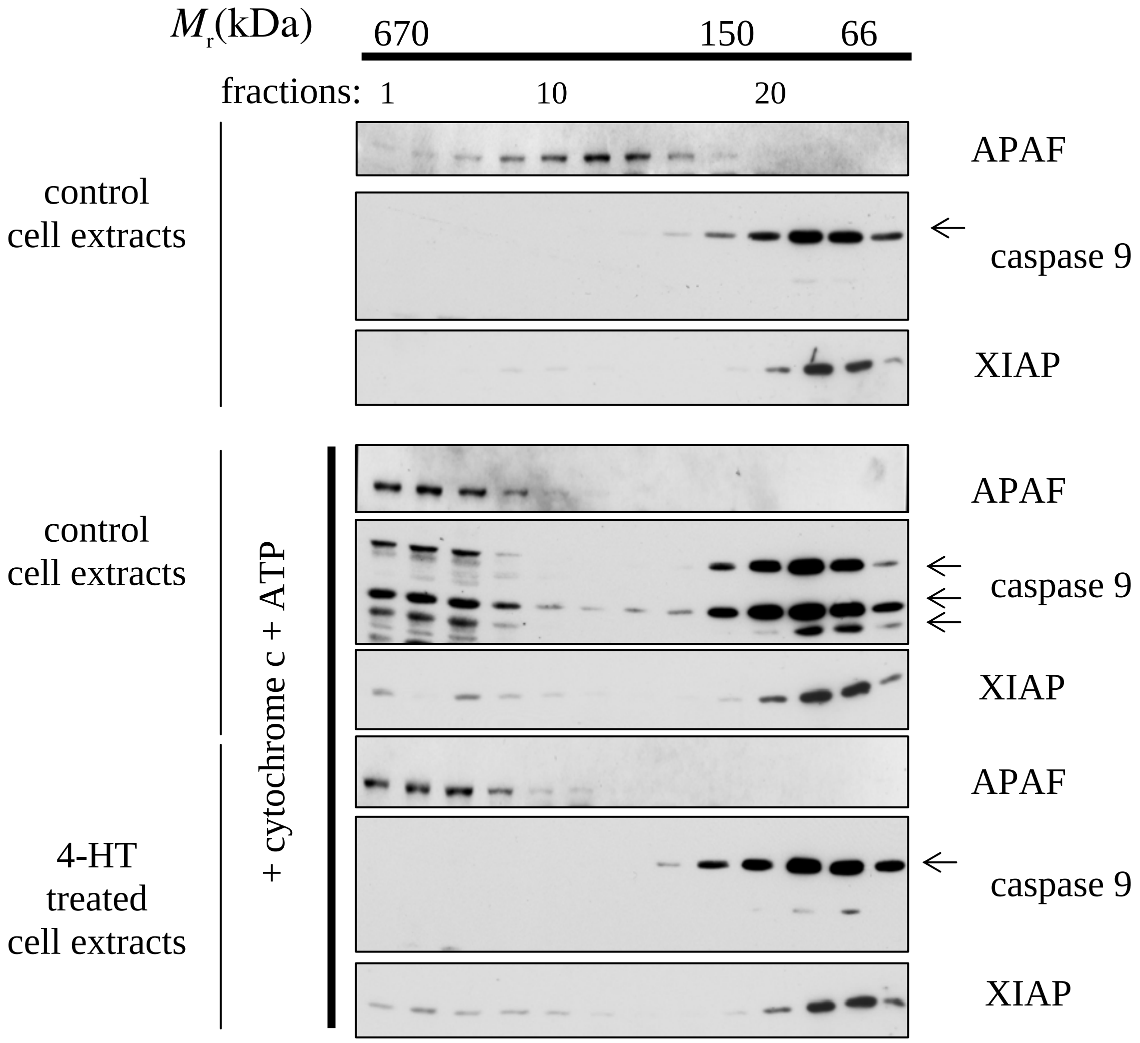

Figure S2: Recruitment of caspase 9 in the apoptosome following in vitro activation of cell extracts with cytochrome c and ATP.

Cell extracts from exponentially growing CCL39-ΔRaf-1:ER cells that were preincubated or not for 9hours with 4-HT were activated as in figure 6 and analysed as in figure 7.
