# Supplementary material for: Raf-1 Activation Prevents Caspase 9 Processing Downstream of Apoptosome Formation
Source: J Signal Transduct. 2010 Oct 14;2011:834948. doi: 10.1155/2011/834948 (PMC3100593; doi:10.1155/2011/834948)
Supplement: Supplementary file 3 [file 834948.f3.pdf]

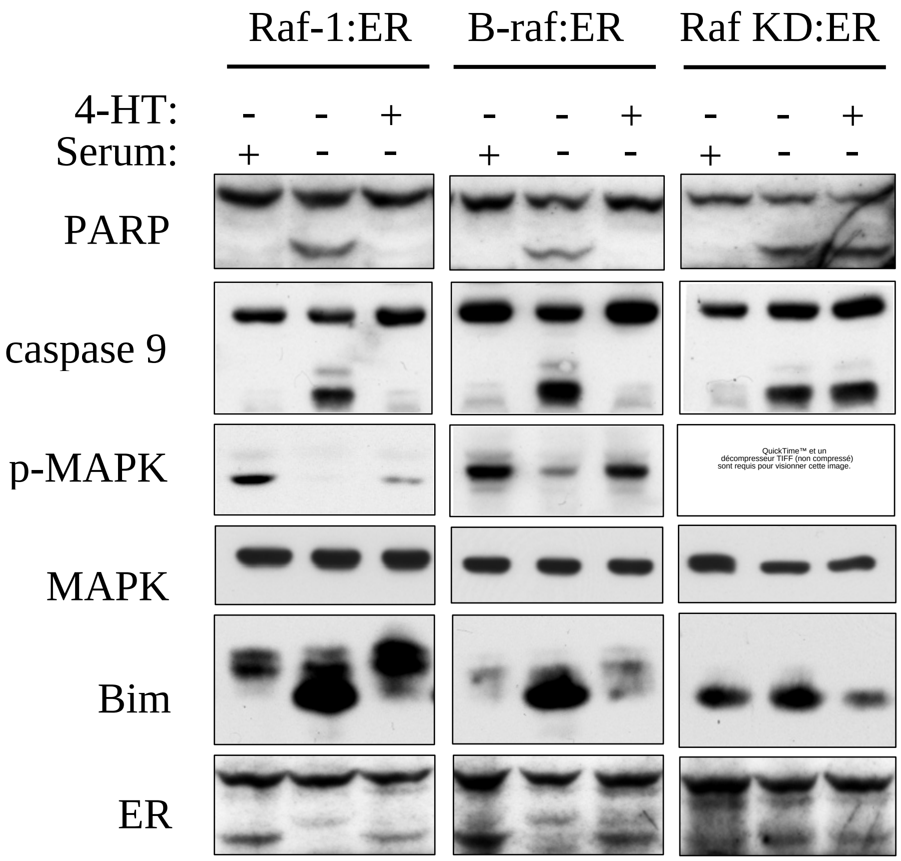

Figure S3: Raf activity is required to prevent caspase 9 cleavage and apoptosis in response to 4-HT.

CCL39 subclones expressing either Raf-1:ER, B-Raf:ER or a catalytically inactive form of Raf-1 (kinase-dead K375W) Raf KD:ER (plasmids were kindly provided by Dr. M. McMahon, University of California San Francisco Cancer Center, San Francisco, CA). were deprived of serum for 24 hours in the presence or absence of 1μM 4-HT. The cell lysates were analyzed by immunoblotting. Cells expressing Raf KD:ER (as indicated by the presence ER, upper band is endogenous, lower band is Raf:ER construct) are unable to activate MAPK (no p-MAPK, no Bim shift) and prevent caspase 9 and PARP cleavage in response to 4-HT.
